# Supplementary material for: Endocytic sorting motif interactions involved in Nef-mediated downmodulation of CD4 and CD3
Source: Nat Commun. 2017 Sep 5;8:442. doi: 10.1038/s41467-017-00481-z (PMC5585231; doi:10.1038/s41467-017-00481-z)
Supplement: Supplementary file 1 — Supplementary Information [file 41467_2017_481_MOESM1_ESM.pdf]

Description of Supplementary Files

File name: Supplementary Information
Description: Supplementary figures, supplementary tables and supplementary note.

File name: Peer review file

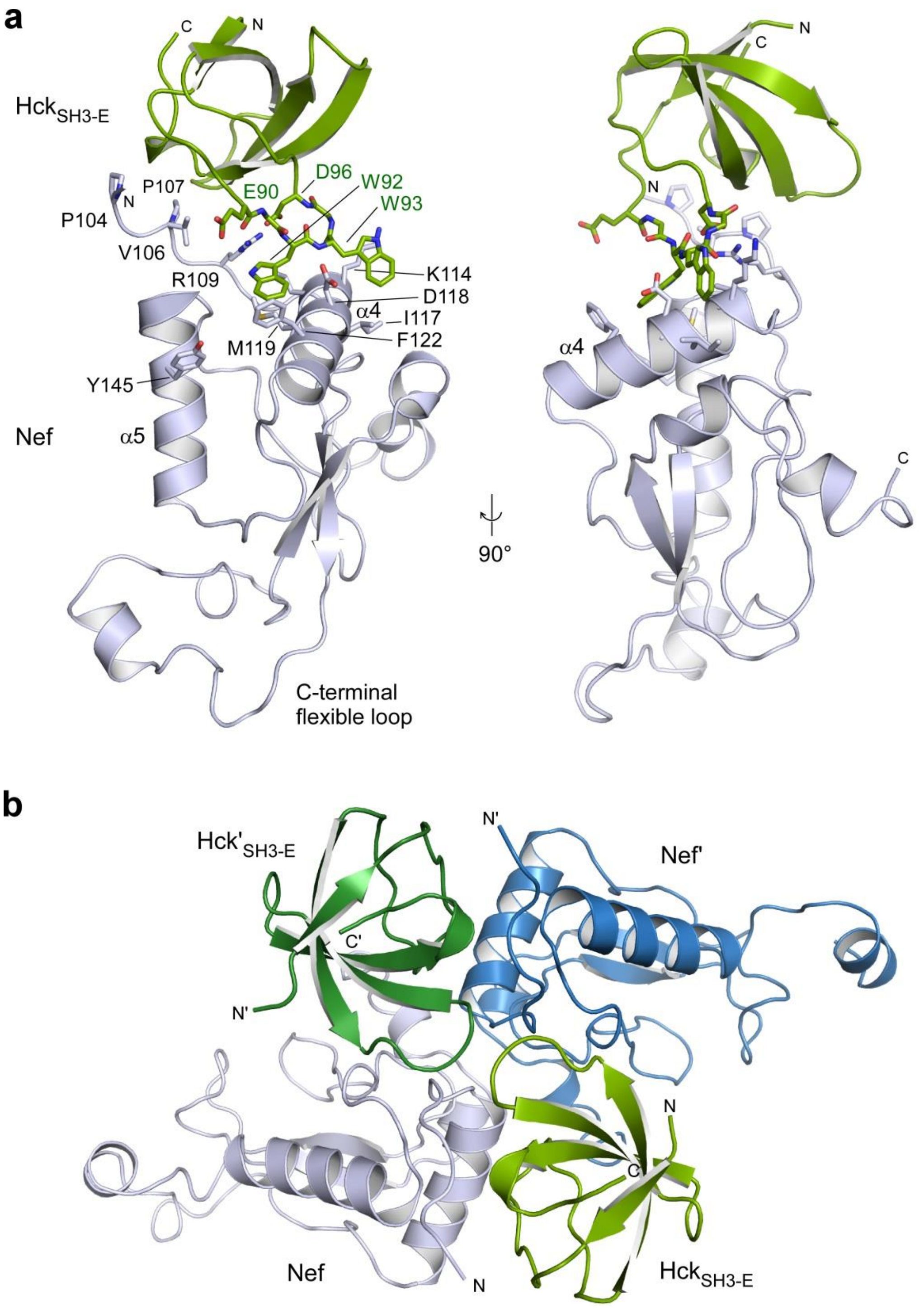

Supplementary Figure 1 Binding of Hck$_{\text{SH3-E}}$ to SIV$_{\text{mac239}}$ Nef and complex assembly. (**a**) Interactions between the Hck$_{\text{SH3-E}}$ domain (green) and SIV$_{\text{mac239}}$ Nef (white/blue). The RT loop sequence of the SH3 domain has been engineered for high affinity binding to Nef and was modified to E$_{90}$GWWG[31]. The two tryptophanes mediate interactions with residues on helix α4 of Nef. The key interactions of the P$_{104}$xVPxR motif of SIV Nef are displayed. The salt bridge formation of R109$_{\text{Nef}}$ to D96$_{\text{Hck}}$ is a major determinant for binding. Important interacting residues are shown in stick representation. (**b**) Assembly of the Nef-Hck heterodimer in the asymmetric unit cell of the crystal structure. Of note, the crystallographic dimer formed by R105 and D123 of Nef$_{\text{NL4-3}}$, corresponding to the conserved residues R137 and D155 in SIV$_{\text{mac239}}$ Nef is not observed in this structure.

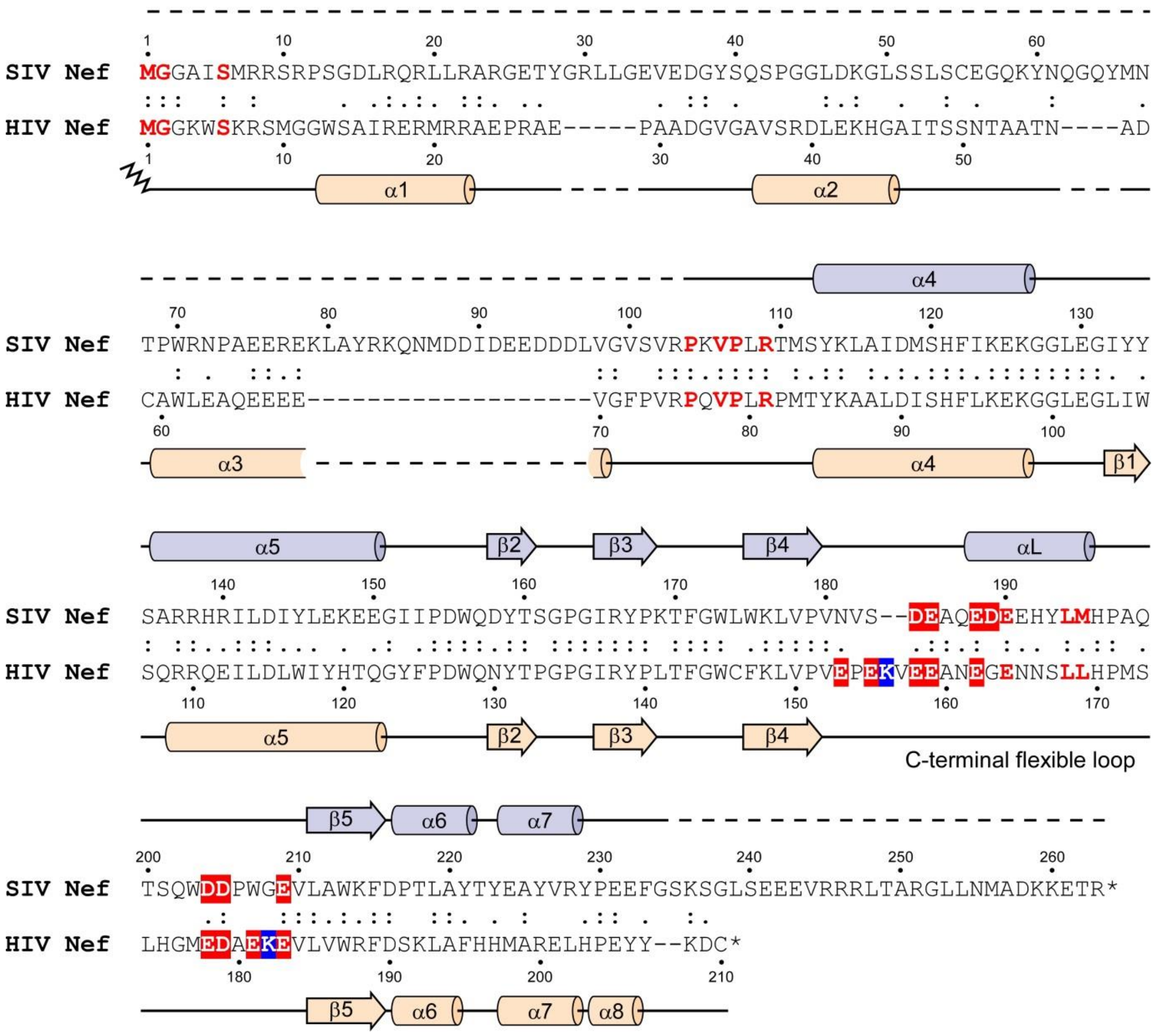

Supplementary Figure 2 Sequence alignment of SIV_{mac239} Nef and HIV-1 Nef_{SF2} and secondary structure display.

The sequences of SIV_{mac239} Nef and HIV-1 Nef_{SF2} share 34.5% identity and 52.9% similarity. Insertions in the SIV allele relative to the HIV-1 sequence are located around position 30, a highly charged stretch of 19 residues (3 basic and 8 acidic residues) from amino acids 79 to 97 preceding the PxxP motif, and 25 residues at the very C-terminus. The three key elements of Nef proteins, the myristoylation motif MGxxxS at the N-terminus, the central PxϕPxR motif, and the dileucine-based sorting motif ExxxLϕ in the C-terminal flexible loop, are conserved and highlighted in red. Charged residues flanking the ExxxLϕ sorting motif in the C-terminal flexible loop are boxed. The secondary structures of SIV_{mac239} Nef determined here (PDB accession code 5NUI) and of HIV-1 Nef_{SF2} (assembled from protein structures 1QA5, 3REA, and 3RBB) are displayed above and below the sequences, respectively.

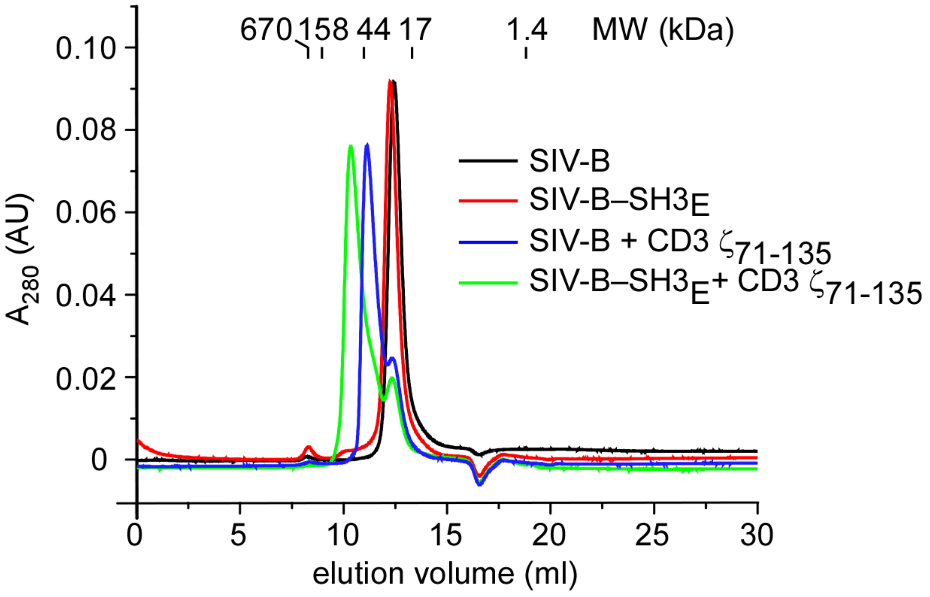

Supplementary Figure 3 Size exclusion chromatography of the tripartite complex between SIV_{mac239} Nef, an SH3 domain, and the cytoplasmic tail of CD3 ζ.

The SIV_{mac239} Nef (66-235) protein, termed SIV-B, eluted as a homogeneous peak at its apparent size of 23.4 kDa. Addition of the SH3 domain from Hck, engineered in the RT loop for optimized binding to SIV Nef, termed $SH3_E$, only slightly increased the size of the complex, similarly as observed before[33]. Addition of CD3 ζ (71-135) containing the two SIV Nef interaction domains (SNID1 and SNID2) led to complex formation with Nef. Likewise, addition of CD3 ζ to the preformed SIV Nef–$SH3_E$ domain complex led to formation of the tripartite complex. The analytical gel filtration was performed using a Superdex S75 (10/300 GL) column (GE Healthcare) on a multicomponent Waters 626 LC system (Waters, MA). Elution profiles were run in 20 mM HEPES (pH 8.0), 100 mM NaCl, and 1 mM TCEP at room temperature.

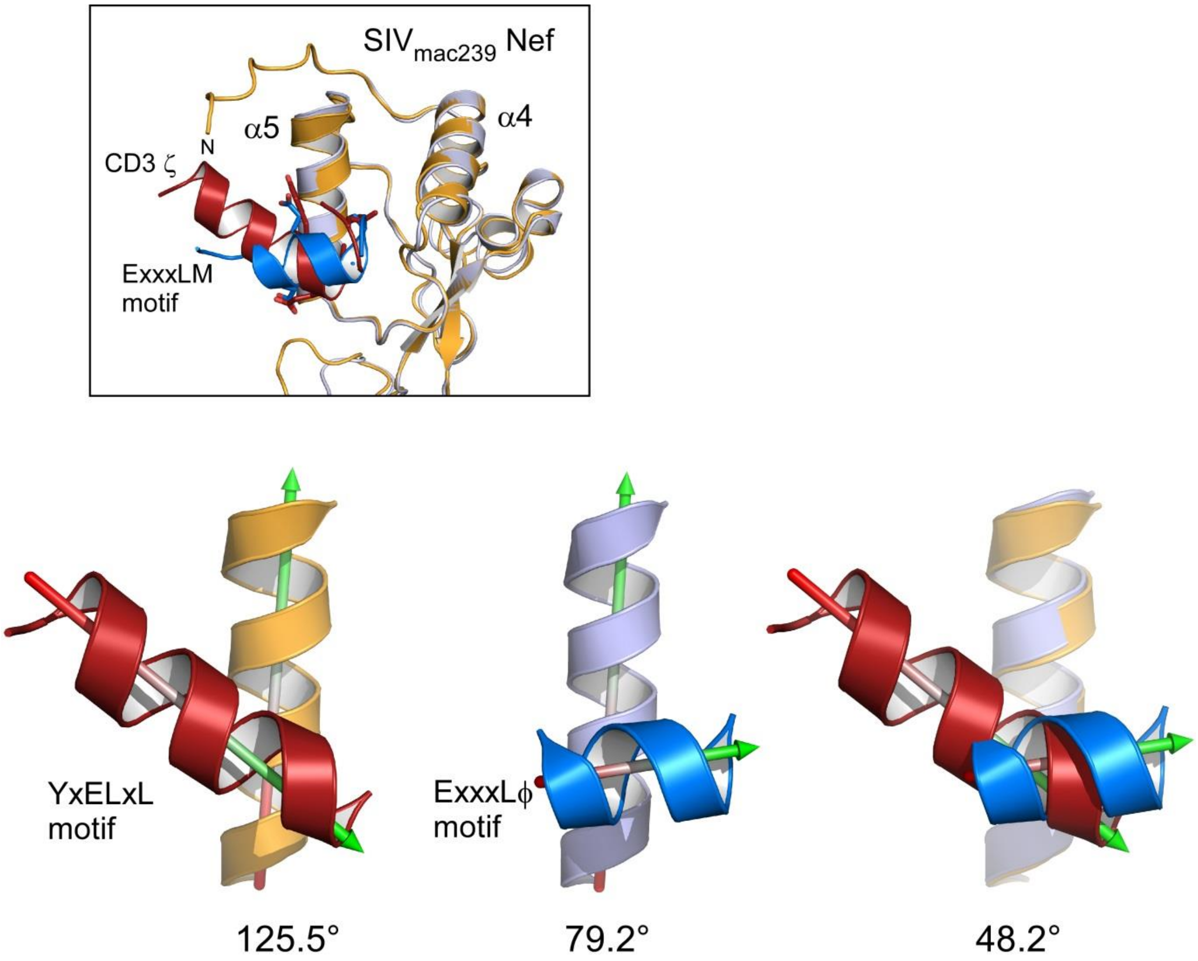

Supplementary Figure 4 Orientation of the sorting motif helices relative to SIV Nef.
The structures of SIV_{mac239} Nef bound to the CD3 ζ complex (PDB accession code 3IK5)[35] or bound to the dileucine-based sorting motif ExxxLM are shown as cartoon model in orange/red and light blue/blue, respectively. The structures were aligned for the Nef core domain (inset). The N- to C-terminal direction of the bound helices of the YxELxL motif (red) and the ExxxLM motif (blue) is similar but the orientation of the helix relative to Nef varies by 48°.

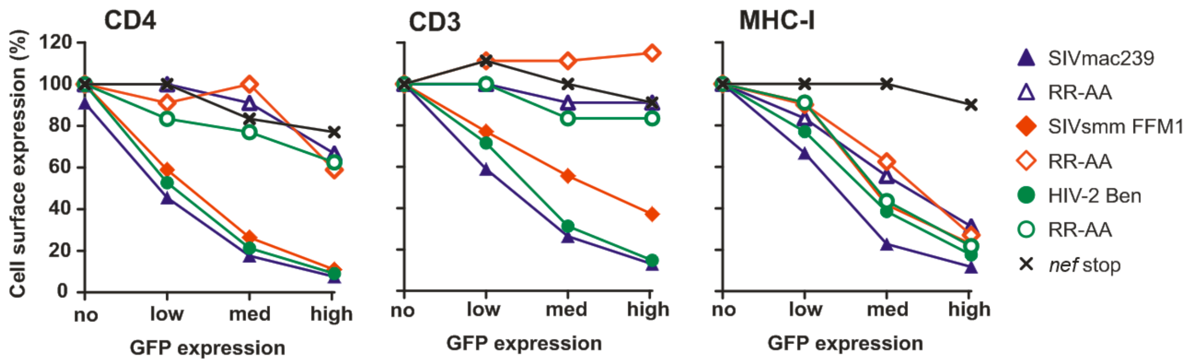

Supplementary Figure 5 Importance of the di-arginine motif for Nef-mediated down-modulation of CD4, CD3, and MHC-I.

Jurkat T cells were transfected with bicistronic vectors coexpressing the indicated *nef* alleles and GFP, and assayed for surface expression of CD4, CD3, and MHC-I by flow cytometry. Receptor surface expression was determined in cells expressing no, low, medium (med), or high levels of GFP as described in the Methods section. The results of one representative experiment are shown.

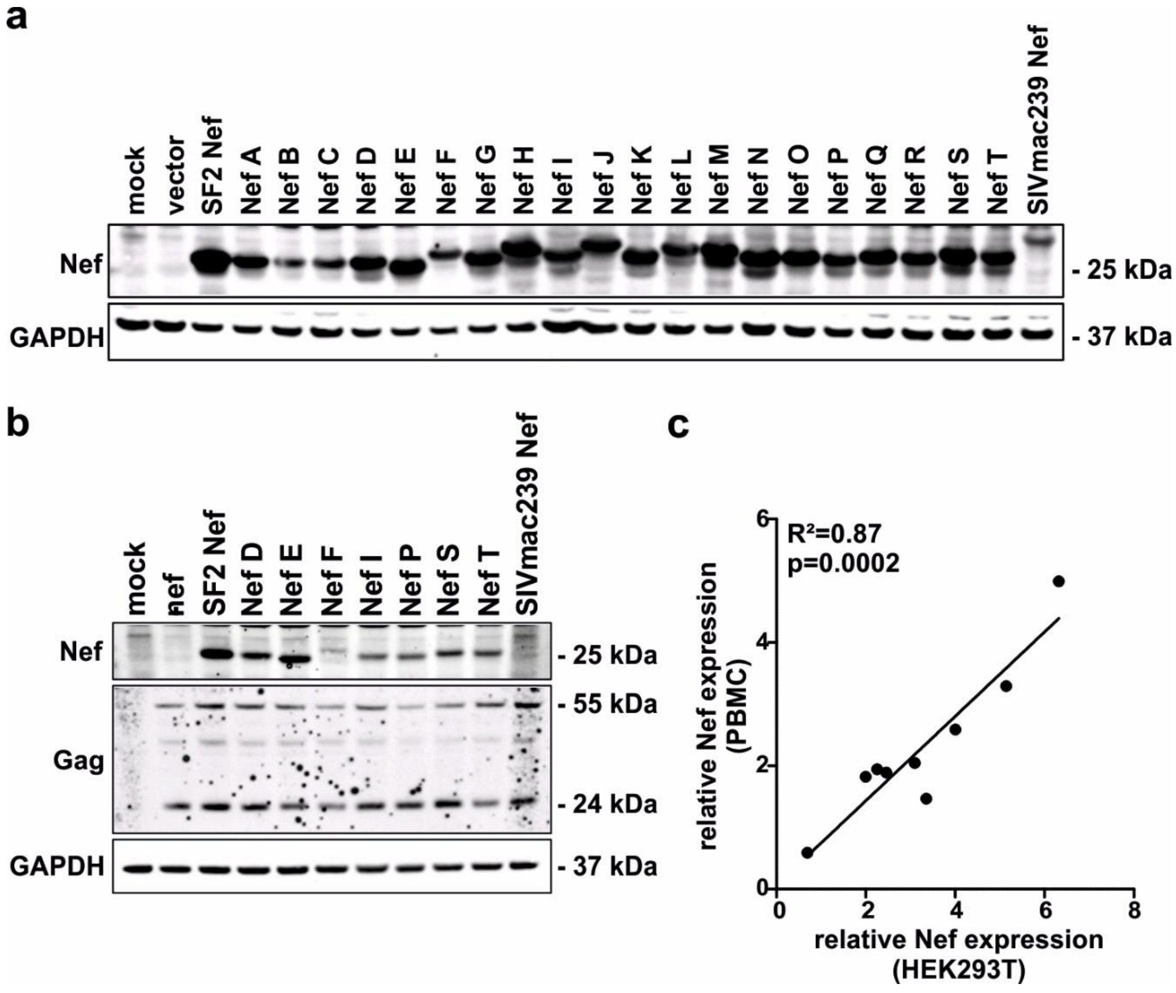

Supplementary Figure 6 Nef expression in transfected HEK293T cells and infected PBMCs.

(**a**) HEK293T cells were transfected with expression vectors for the indicated Nef proteins. Two days post transfection cells were lysed and analyzed by Western blotting. Nef was detected using a rabbit antiserum directed against HIV-1 Nef. GAPD1 served as loading control. (**b**) PBMCs were infected with HIV-1 SF2 constructs expressing the indicated Nef proteins. Three days post infection, cells were analyzed by Western blotting as described for (a). HIV-1 Gag was detected using an antiserum against HIV-1 p24. (**c**) Nef expression levels in transfected HEK293T cells (n=1) and infected PBMCs (n=2) were quantified and normalized to GAPDH and HIV-1 Gag, respectively. Pearson‘s correlation coefficient was calculated.

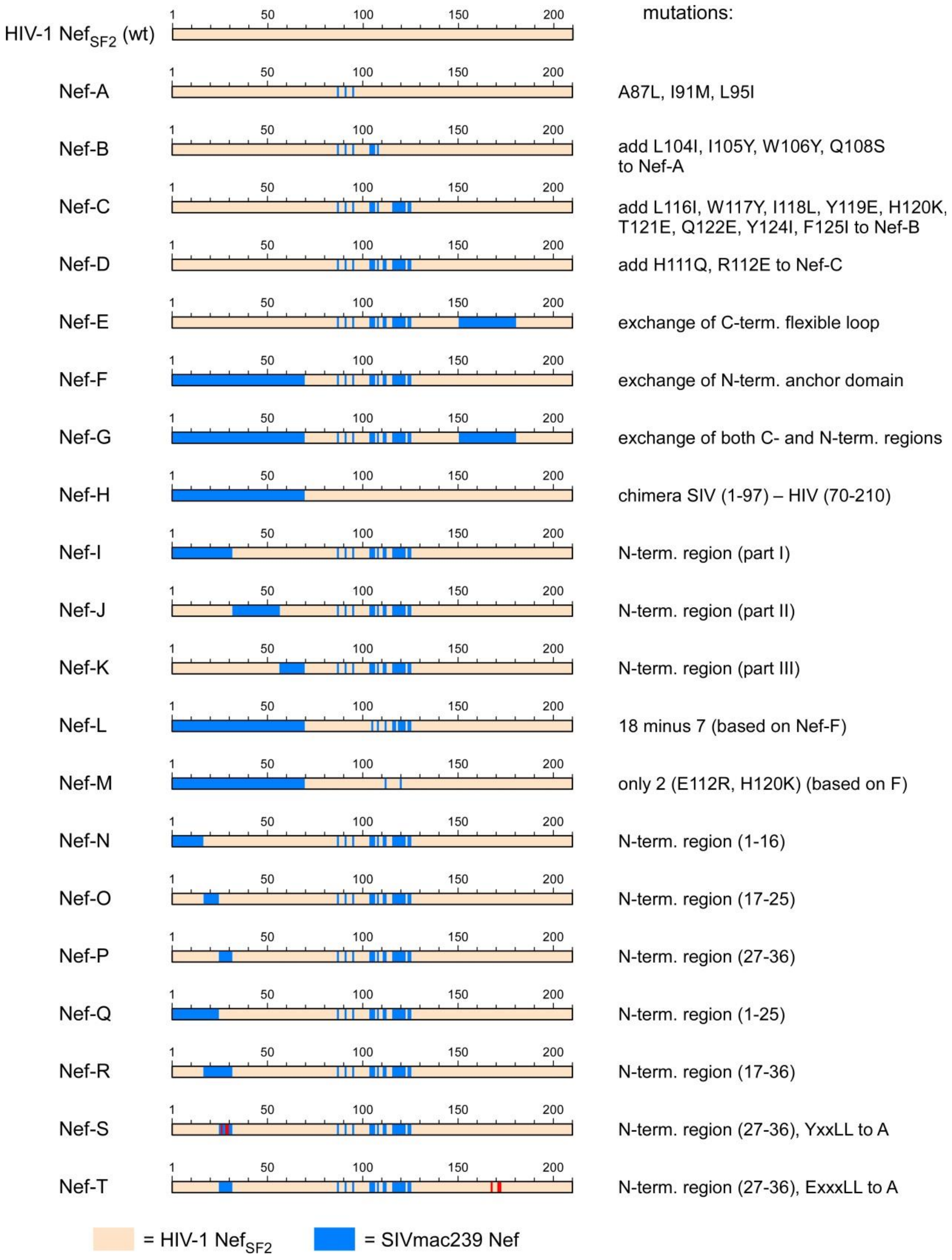

Supplementary Figure 7 HIV-1 Nef gain-of-function mutations for CD3 ζ binding.
Based on the sequence of HIV-1 Nef$_{SF2}$ of 210 amino acids, single point mutations and sequence exchanges were generated for the acquisition of CD3 down-regulation, using the sequence of SIV$_{mac239}$ Nef as template for gain-of-function. First, point mutations were introduced in the hydrophobic crevice of Nef ranging from residues 80 to 130. Next, the N-terminal anchor domain and C-terminal flexible loop section were exchanged. These sequence stretches were further optimized to determine the minimally required regions. Finally, the YxxL and ExxxLL motifs were mutated to alanines to unravel the functions of these sorting signals for Nef mediated endocytosis.

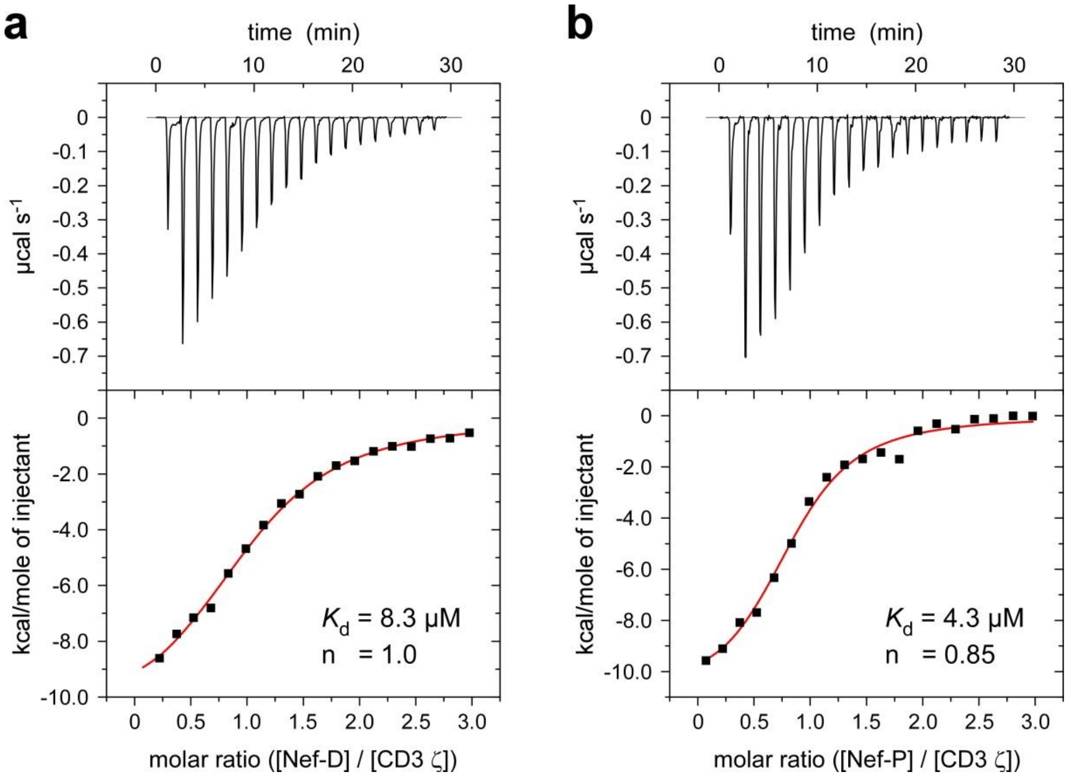

Supplementary Figure 8 ITC measurements between Nef gain-of-function variants D and P and CD3 ζ ITAM motifs.

(**a**) Nef-D and (**b**) Nef-P proteins were expressed as recombinant proteins with domain boundaries 23-210, C210A. Both proteins contain the 18 mutations in the α4/α5 core domain of Nef. Nef-P contains in addition the N-terminal YxxL motif. The affinity of Nef-P for the second ITAM motif of CD3 ζ is only about 2-fold higher than that of Nef-D. The significantly increased ability to down-regulate CD3 by Nef-P compared to Nef-D is therefore not a result of much better binding to CD3, but rather supposed to be due to the improved interaction with the adaptor protein machinery.

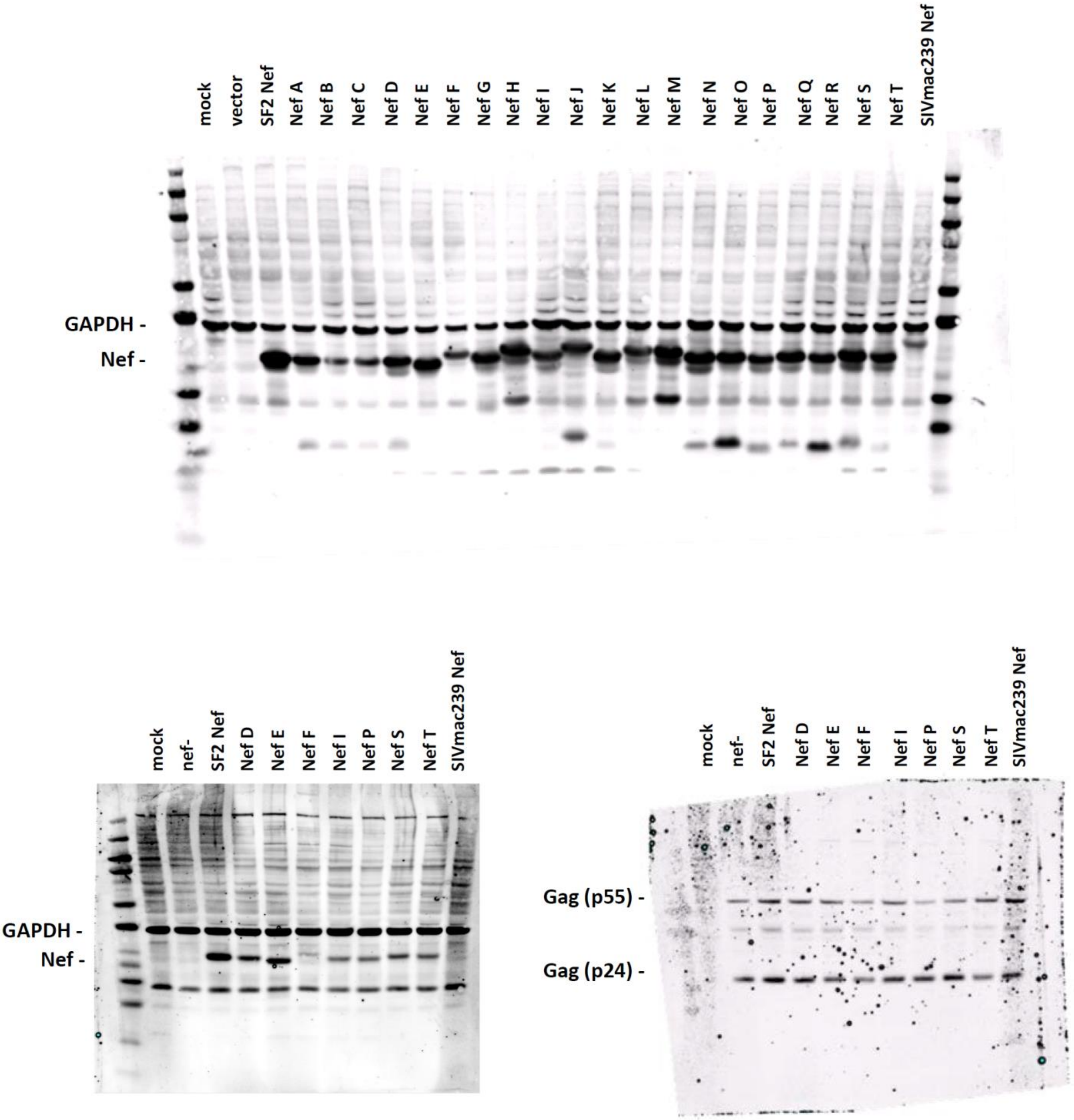

Supplementary Figure 9 Uncropped images of the Western blot results shown in Supplementary Figure 6.

Supplementary Table 1 Thermodynamic parameters of Nef–CD3 ζ interactions determined by isothermal titration calorimetry[1]

titration scheme[2]	K_D (μM)	ΔH (kcal/mol)	$T\Delta S$ (kcal/mol)	molar ratio n
SIV_{mac239} Nef to CD3 ζ SNID2	2.6 ± 0.33	-13.97 ± 0.17	-6.35	1.07
HIV-1 Nef_{SF2} to CD3 ζ SNID2	–	–	–	–
Nef (H-to-S) to CD3 ζ SNID2	4.4 ± 0.43	-13.34 ± 0.41	-6.02	0.86
Nef (15m) to CD3 ζ SNID2	10.8 ± 0.41	-9.09 ± 0.36	-2.32	0.39
Nef (13m) to CD3 ζ SNID2	15.9 ± 4.79	-10.83 ± 0.36	-4.29	0.90
Nef-D to CD3 ζ SNID2	8.33 ± 0.74	-11.26 ± 0.37	-4.32	1.03
Nef-P to CD3 ζ SNID2	4.27 ± 0.74	-11.18 ± 0.49	-3.84	0.82

[1] all measurements were performed at 25°C.
[2] SIV_{mac239} Nef encompassed residues 66-235, HIV-1 Nef_{SF2} contained residues 45-210, and Nef-D and Nef-P residues 23-210. The 22-mer SNID2 peptide contained amino acids 114-135 of human CD3 ζ.

Supplementary Table 2 Oligonucleotides used in this study for PCR product generation

SIVmac239 Nef codon optimization for E.coli expression

SIVmac239 Nef 1+; start NcoI (1+)

Nef94 5’-CATGCCATGGGTGGAGCTATTTCCATGCGTCGGTCCCGGCCGT-3’

reverse (77-)

Nef95 5’-CGCTCTTCAGCTGGGTTACGCCAAGGAGTATTCAT-3’

forward (66+)

Nef96 5-ATGAATACTCCTTGGCGTAACCCAGCTGAAGAGCGAGAA-3’

reverse (145-)

Nef97 5’-GTATATGTCTAAGATTCGATGCCGACGTGCACTGTA-3’

forward (133+)

Nef98 5’-TATTACAGTGCACGTCGGCATCGAATCTTAGAC-3’

reverse (249-)

Nef99 5’-GGTCAGCCGACGTCGAACCTCTTCCTCTGACAGGCCTGA-3’

forward (238+)

Nef100 5’-CCTGTCAGAGGAAGAGGTTCGACGTCGGCTGACCGCACGAGGC-3’

SIV mac239 Nef (263-, HindIII, w/o stop, for C-term. His-tag, in frame with pET-23b)

Nef101 5’-CCCAAGCTTGCGAGTTTCCTTCTTGTCAGCCATGTT-3’

SIVmac239 Nef constructs for biochemical and structural studies

SIVmac239 Nef 1+; start NcoI (1+)

Nef94 5’-CATGCCATGGGTGGAGCTATTTCCATGCGTCGGTCCCGGCCGT-3’

SIVmac239 Nef 66+; N67A, start: NcoI (66+)

Nef132 5’-CATGCCATGGCTACTCCTTGGCGTAACCCAGCTGAAG-3’

SIVmac239 Nef 263-; stopp EcoRI (263-)

Nef133 5’-CGGAATTCTCAGCGAGTTTCCTTCTTGTCAGCCATGTT-3’

SIVmac239 Nef 235-; stopp EcoRI (235-)

Nef135 5’-CGGAATTCTCAGCTTCCAAACTCTTCTGGGTATCTAAC-3’

SIVmac239 Nef 98+; start: NcoI, (98+), L97M GAM-VGVSV…

Nef192 5'-CATGCCATGGTAGGGGTATCAGTGAGGCCAAAAGTTCC-3'

SIV mac239 Nef 87+; start: NcoI, (87+), GA-MDDIDE…

Nef200 5'-CATGCCATGGATGATATAGATGAGGAAGATGATGAC-3'

SF2 Nef constructs for the analysis of CD3 zeta binding (gain-of-function)

Nef-SF2 L104I, forward + reverse

Nef196 5’-AAAGAAAAGGGGGGACTGGAAGGGATCATTTGGTCC-3’

Nef197 5’-CTCTTGACGGCGTTGGGACCAAATGATCCCTTCCAG-3’

Nef-SF2 W117Y, forward + reverse

Nef198 5’-CGCCGTCAAGAGATCCTTGATCTGTACATCTACCAC-3’

Nef199 5’-GAAGTAGCCTTGTGTGTGGTAGATGTACAGATCAAG-3’

Nef-SF2 triple mutant (A87L, I91M, L95I), forward + reverse

Nef204 5’-TACAAGTTAGCTTTAGATATGAGCCACTTTATAAAAGAAAAG-3’

Nef205 5’-TATAAAGTGGCTCATATCTAAAGCTAACTTGTAAGT-3’

Nef-SF2 18-mer mutant, forward + reverse

Nef210 5’-CCGTCATCGGATCCTTGATATATACCTCGAAAAGGAAGAAGGCATCATCCCTG-3’

Nef211 5’-TTTCGAGGTATATATCAAGGATCCGATGACGGCGTGCGGAGTAATAGATCCCT-3’

Nef-SF2 H120,T121 to KE, forward + reverse

Nef212 5’-ATCCTTGATCTGTACATCTACAAGGAACAAGGCTAC-3’

Nef213 5’-CCAATCAGGGAAGTAGCCTTGTTCCTTGTAGATGTA-3’

Nef-SF2 13-mer mutant; forward + reverse

Nef219 5’-CCGTCAAGAGATCCTTGATATATACCTCTACAAGGAAGAAGGCATCTTCCCTG-3’

Nef220 5’-TGTAGAGGTATATATCAAGGATCTCTTGACGGCGTTGGGAGTAA-3’

Nef-SF2 11-mer mutant; forward + reverse

Nef221 5’-CCGTCAAGAGATCCTTGATATATACCTCTACAAGGAAGAAGGCTACTTCCCTG-3’

Nef222 5’-TGTAGAGGTATATATCAAGGATCTCTTGACGGCGTTGGGACCAATAGAT-3’

Gain-of-function mutations in Nef IRES GFP construct

Nef-SF2 1+; start: Xba1 (1+)
Nef249 5'-ACCTATCTAGATACAATATGGGTGGCAAGTGGTCAAAACGT-3'
Nef-SF2 210-; stopp: Mlu1 (210-)
Nef250 5'-GTCCCTACGCGTCAGCGAGTTTCCTTCTTG-3'
Nef-SF2 210-; stopp: Mlu1 (210-)
Nef251 5'-GTCCCTACGCGTTCAGCAGTCTTTGTAGTACTCCGG-3'

Gain-of-function mutations in Nef IRES GFP construct SF2 Nef with SIVmac239 flexible loop or SIVmac239 N-terminal domain

Nef-SF2 147+, for fusion with N-terminal Nef-SF2 constructs
forward-1
Nef263 5'-AAGCTAGTACCAGTTAATGTATCAGATGAGGCACAGGAGGATGAGGAG-3'
reverse-2
Nef264 5'-CTGGGAAGTTTGAGCTGGATGCATTAAATAATGCTCCTCATCCTCCTGTGCCT-3'
forward-3
Nef265 5'-ATCCAGCTCAAACTTCCCAGTGGGATGACCCTTGGGGAGAAGTGTTAGTGTGG-3'
reverse-4
Nef266 5'-CATGTGATGAAATGCTAGTTTGCTGTCAAACCTCCACACTAACACTTCTCCCC-3'
forward-5
Nef267 5'-AACTAGCATTTCATCACATGGCCCGAGAGCTGCATCCGGAGTACTACAAAGAC-3'
reverse-6, stopp: Mlu1 (210-)
Nef268 5'-GTCCCTACGCGTTCAGCAGTCTTTGTAGTACTCCGGATG-3'
reverse primer for N-terminal part
Nef269 5'-CTCATCTGATACATTAACTGGTACTAGCTTGAAGCACCATCC-3'
mac239-Nef 1+ for SIV/HIV chimeras, start: Xba1 (1+)
Nef270 5'-ACCTATCTAGATACAATATGGGTGGAGCTATTTCCATGCGTCGG-3'
mac239-Nef 97-, reverse
Nef271 5'-GACTGGAAAACCCACCAAGTCATCATCTTCCTCATCTATATC-3'
SF2-Nef 70+, forward, for mega primer PCR
Nef272 5'-GAAGATGATGACTTGGTGGGTTTTCCAGTCAGACCTCAGGTA-3'
mac239-Nef 36-, reverse. for mega primer PCR
Nef275 5'-TGCTCCCACCCCATCTTCCACCTCTCCTAAGAGTCTCCCATA-3'
SF2-Nef 32+, forward, for mega primer PCR
Nef276 5'-TTAGGAGAGGTGGAAGATGGGGTGGGAGCAGTATCTCGAGAC-3'
SF2-Nef 33-, reverse, for mega primer PCR
Nef277 5'-TGGGGATTGCGAGTACCCATCTGCTGCTGGCTCAGCTCGTGG-3'
mac239-Nef 39+, forward, for mega primer PCR
Nef278 5'-CCAGCAGCAGATGGGTACTCGCAATCCCCAGGAGGATTAGAC-3'
mac239-Nef 87-, reverse
Nef279 5'-CCAGGCACAATCAGCATTTTGTTTTCTGTATGCTAATTTTTC-3'
SF2-Nef 57+, forward (57+)
Nef280 5'-TACAGAAAACAAAATGCTGATTGTGCCTGGCTAGAAGCACAA-3'
SF2-Nef 56-, reverse (56-)
Nef281 5'-ATCATCTTCCTCATCTATATCATCCATATTAGTAGCTGCTGTATTGCTACTTGTGATTGC-3'
SF2-Nef 70+, forward
Nef282 5'-GATATAGATGAGGAAGATGATGACTTGGTGGGTTTTCCAGTCAGACCTCAGGTACCTTTA-3'

Gain-of-function mutation in Nef IRES GFP

Nef-SF2 L; forward + reverse
Nef291 5'-AGACAACGGATCCTTGATATCTACATCGAGAAGGAAGAAGGCATCATCCCTG-3'
Nef292 5'-TTCTCGATGTAGATATCAAGGATCCGTTGTCTTCTTGCGGACCAATATAGCC-3'
SF2-Nef M; forward + reverse
Nef293 5'-CAACGGATCCTTGATCTGTGGATCTACAAGACACAA-3'
Nef294 5'-GTCTTGTAGATCCACAGATCAAGGATCCGTTGTCTT-3'
SF2-Nef N, forward + reverse
Nef295 5'-CCGTCTGGAGATCTGAGGGAAAGAATGAGACGAGCTGAGCCA-3'
Nef296 5'-TCTCATTCTTTCCCTCAGATCTCCAGACGGCCTGGACCGACG-3'

SF2-Nef P, on Nef-I template; forward
Nef297 5'-ATGAGACGAGCTGAGACTTATGGGAGACTCTTAGGAGAGGTG-3'
SF2-Nef P, on Nef-D template; reverse
Nef298 5'-GAGTCTCCCATAAGTCTCAGCTCGTCTCATTCTTTCCCTTAT-3'
SF2-Nef O and Q, on Nef-D template; forward
Nef299 5'-TTGCGGGCGCGTGGGGAGCCACGAGCTGAGCCAGCAGCAGAT-3'
SF2-Nef Q on Nef-I template; Nef O on Nef-R template, reverse
Nef300 5'-CTCAGCTCGTGGCTCCCCACGCGCCCGCAAGAGTCTCTGTCG-3'
SF2-Nef R, on Nef-I template; forward
Nef301 5'-GGATGGTCTGCTATACGACAGAGACTCTTGCGGGCGCGTGGG-3'
SF2-Nef R, on Nef-D template; reverse
Nef302 5'-CAAGAGTCTCTGTCGTATAGCAGACCATCCACCCATACTACG-3'
SF2-Nef S based on template Nef-P; forward + reverse
Nef322 5'-GAGCCGCAGGAGAGGTGGAAGATGG-3'
Nef323 5'-TCCCAGCAGTCTCAGCTCGTCTCATTC-3'
SF2-Nef T based on template Nef-P; forward + reverse
Nef324 5'-AGCGCGGCACACCCTATGAGCCTGCA-3'
Nef325 5'-GTTGTTCGCTCCTTCATTGGCCTCTTC-3'

Nef-SF2 and Nef-P in comparison for CD3-zeta for ITC
SF2-Nef-P 23+ (for GAM-23-210, C210A); start: NcoI (23+)
Nef320 5'-CATGCCATGGCTGAGACTTATGGGCGACTCTTAGGAGAG-3'
SF2-Nef 27+ (for GAM-27-210, C210A); start: NcoI (27+)
Nef321 5'-CATGCCATGGCTGAGCCAGCAGCAGATGGGGTGGGAGCA-3'

Hck-SH3 domain with RT-loop change EAIHHE to EGWWG
start: NcoI (79+); forward
Nef148 5'-CATGCCATGGAGGACATCATCGTGGTTGCCCTGTATGATTACGAGGGCTGGTGG GGAGACCTCAGCTTCCAGAAGGGG-3'
Hck-SH3, (138-); reverse, stop: EcoR1
Nef149 5'-CGGAATTCTCAAGAGTCAACGCGGGCGACATAGTT-3'

CD3 zeta chain
human zeta chain (59+); start: NcoI
Nef153 5'-CATGCCATGGCAGACGCCCCCGCGTACCAGCAGGGC-3'
human zeta chain (71+); start: NcoI
Nef173 5'-CATGCCATGGCGCTCTATAACGAGCTCAATCTTGGACGA-3'
human zeta chain (135-), stopp, EcoRI
Nef174 5'-CGGAATTCTCAGCCGCGCCGGCGCTCGCCTTTCATCCCA-3'

Supplementary Note 1

HIV-1 Nef-SF2 (AC: P03407)
MGGKWSKRSMGGWSAIRERMRRAEPRAEPAADGVGAVSRDLEKHGAITSSNTAATNADCA
WLEAQEEEEVGFPVRPQVPLRPMTYKAALDISHFLKEKGGLEGLIWSQRRQEILDLWIYH
TQGYFPDWQNYTPGPGIRYPLTFGWCFKLVPVEPEKVEEANEGENNSLLHPMSLHGMEDA
EKEVLVWRFDSKLAFHHMARELHPEYYKDC

SIVmac239 Nef (AC: M33262)
MGGAISMRRSRPSGDLRQRLLRARGETYGRLLGEVEDGYSQSPGGLDKGLSSLSCEGQKY
NQGQYMNTPWRNPAEEREKLAYRKQNMDDIDEEDDDLVGVSVRPKVPLRTMSYKLAIDMS
HFIKEKGGLEGIYYSARRHRILDIYLEKEEGIIPDWQDYTSGPGIRYPKTFGWLWKLVPV
NVSDEAQEDEEHYLMHPAQTSQWDDPWGEVLAWKFDPTLAYTYEAYVRYPEEFGSKSGLS
EEEVRRRLTARGLLNMADKKETR

Nef-A
MGGKWSKRSMGGWSAIRERMRRAEPRAEPAADGVGAVSRDLEKHGAITSSNTAATNADCA
WLEAQEEEEVGFPVRPQVPLRPMTYK**L**ALD**M**SHF**I**KEKGGLEGLIWSQRRQEILDLWIYH
TQGYFPDWQNYTPGPGIRYPLTFGWCFKLVPVEPEKVEEANEGENNSLLHPMSLHGMEDA
EKEVLVWRFDSKLAFHHMARELHPEYYKDC

Nef-B
MGGKWSKRSMGGWSAIRERMRRAEPRAEPAADGVGAVSRDLEKHGAITSSNTAATNADCA
WLEAQEEEEVGFPVRPQVPLRPMTYK**L**ALD**M**SHF**I**KEKGGLEG**IYY**S**A**RRQEILDLWIYH
TQGYFPDWQNYTPGPGIRYPLTFGWCFKLVPVEPEKVEEANEGENNSLLHPMSLHGMEDA
EKEVLVWRFDSKLAFHHMARELHPEYYKDC

Nef-C
MGGKWSKRSMGGWSAIRERMRRAEPRAEPAADGVGAVSRDLEKHGAITSSNTAATNADCA
WLEAQEEEEVGFPVRPQVPLRPMTYK**L**ALD**M**SHF**I**KEKGGLEG**IYY**S**A**RRQEILD**IYLEK**
EEG**II**PDWQNYTPGPGIRYPLTFGWCFKLVPVEPEKVEEANEGENNSLLHPMSLHGMEDA
EKEVLVWRFDSKLAFHHMARELHPEYYKDC

Nef-D
MGGKWSKRSMGGWSAIRERMRRAEPRAEPAADGVGAVSRDLEKHGAITSSNTAATNADCA
WLEAQEEEEVGFPVRPQVPLRPMTYK**L**ALD**M**SHF**I**KEKGGLEG**IYY**S**A**RR**HR**ILD**IYLEK**
EEG**II**PDWQNYTPGPGIRYPLTFGWCFKLVPVEPEKVEEANEGENNSLLHPMSLHGMEDA
EKEVLVWRFDSKLAFHHMARELHPEYYKDC

Nef-E
MGGKWSKRSMGGWSAIRERMRRAEPRAEPAADGVGAVSRDLEKHGAITSSNTAATNADCA
WLEAQEEEEVGFPVRPQVPLRPMTYK**L**ALD**M**SHF**I**KEKGGLEG**IYY**S**A**RR**HR**ILD**IYLEK**
EEG**II**PDWQNYTPGPGIRYPLTFGWCFKLVPV**NVSDEAQEDEEHYLMHPAQTSQWDDPWG**
EVLVWRFDSKLAFHHMARELHPEYYKDC

Nef-F
MGGAISMRRSRPSGDLRQRLLRARGETYGRLLGEVEDGYSQSPGGLDKGLSSLSCEGQKY
NQGQYMNTPWRNPAEEREKLAYRKQNMDDIDEEDDDLVGFPVRPQVPLRPMTYK**L**ALD**M**S
HF**I**KEKGGLEG**IYY**S**A**RR**HR**ILD**IYLEKEE**G**II**PDWQNYTPGPGIRYPLTFGWCFKLVPV
EPEKVEEANEGENNSLLHPMSLHGMEDAEKEVLVWRFDSKLAFHHMARELHPEYYKDC

Nef-G
MGGAISMRRSRPSGDLRQRLLRARGETYGRLLGEVEDGYSQSPGGLDKGLSSLSCEGQKY
NQGQYMNTPWRNPAEEREKLAYRKQNMDDIDEEDDDLVGFPVRPQVPLRPMTYK**L**ALD**M**S
HF**I**KEKGGLEG**IYY**S**A**RR**HR**ILD**IYLEKEE**G**II**PDWQNYTPGPGIRYPLTFGWCFKLVPV
NVSDEAQEDEEHYLMHPAQTSQWDDPWGEVLVWRFDSKLAFHHMARELHPEYYKDC

Nef-H

MGGAISMRRSRPSGDLRQRLLRARGETYGRLLGEVEDGYSQSPGGLDKGLSSLSCEGQKY
NQGQYMNTPWRNPAEEREKLAYRKQNMDDIDEEDDDLVGFPVRPQVPLRPMTYKAALDIS
HFLKEKGGLEGLIWSQRRQEILDLWIYHTQGYFPDWQNYTPGPGIRYPLTFGWCFKLVPV
EPEKVEEANEGENNSLLHPMSLHGMEDAEKEVLVWRFDSKLAFHHMARELHPEYYKDC

Nef-I

MGGAISMRRSRPSGDLRQRLLRARGETYGRLLGEVEDGVGAVSRDLEKHGAITSSNTAAT
NADCAWLEAQEEEEVGFPVRPQVPLRPMTYK**L**ALD**M**SHF**I**KEKGGLEG**IYY**S**A**RR**HR**ILD
IYLEKEEG**II**PDWQNYTPGPGIRYPLTFGWCFKLVPVEPEKVEEANEGENNSLLHPMSLH
GMEDAEKEVLVWRFDSKLAFHHMARELHPEYYKDC

Nef-J

MGGKWSKRSMGGWSAIRERMRRAEPRAEPAADG**YSQSPGGLDKGLSSLSCEGQKYNQGQY**
MNTPWRNPAEEREKLAYRKQNADCAWLEAQEEEEVGFPVRPQVPLRPMTYK**L**ALD**M**SHF**I**
KEKGGLEG**IYY**S**A**RR**HR**ILD**IYLEKEE**G**II**PDWQNYTPGPGIRYPLTFGWCFKLVPVEPE
KVEEANEGENNSLLHPMSLHGMEDAEKEVLVWRFDSKLAFHHMARELHPEYYKDC

Nef-K

MGGKWSKRSMGGWSAIRERMRRAEPRAEPAADGVGAVSRDLEKHGAITSSNTAATN**MDDI**
DEEDDDLVGFPVRPQVPLRPMTYK**L**ALD**M**SHF**I**KEKGGLEG**IYY**S**A**RR**HR**ILD**IYLEKEE**
G**II**PDWQNYTPGPGIRYPLTFGWCFKLVPVEPEKVEEANEGENNSLLHPMSLHGMEDAEK
EVLVWRFDSKLAFHHMARELHPEYYKDC

Nef-L

MGGAISMRRSRPSGDLRQRLLRARGETYGRLLGEVEDGYSQSPGGLDKGLSSLSCEGQKY
NQGQYMNTPWRNPAEEREKLAYRKQNMDDIDEEDDDLVGFPVRPQVPLRPMTYKAALDIS
HFLKEKGGLEGL**Y**WS**A**RRQ**R**ILD**IY**I**EKEE**G**II**PDWQNYTPGPGIRYPLTFGWCFKLVPV
EPEKVEEANEGENNSLLHPMSLHGMEDAEKEVLVWRFDSKLAFHHMARELHPEYYKDC

Nef-M

MGGAISMRRSRPSGDLRQRLLRARGETYGRLLGEVEDGYSQSPGGLDKGLSSLSCEGQKY
NQGQYMNTPWRNPAEEREKLAYRKQNMDDIDEEDDDLVGFPVRPQVPLRPMTYKAALDIS
HFLKEKGGLEGLIWSQRRQ**R**ILDLWIY**K**TQGYFPDWQNYTPGPGIRYPLTFGWCFKLVPV
EPEKVEEANEGENNSLLHPMSLHGMEDAEKEVLVWRFDSKLAFHHMARELHPEYYKDC

Nef-N

MGGAISMRRSRPSGDLRERMRRAEPRAEPAADGVGAVSRDLEKHGAITSSNTAATNADCA
WLEAQEEEEVGFPVRPQVPLRPMTYK**L**ALD**M**SHF**I**KEKGGLEG**IYY**S**A**RR**HR**ILD**IYLEK**
EEG**II**PDWQNYTPGPGIRYPLTFGWCFKLVPVEPEKVEEANEGENNSLLHPMSLHGMEDA
EKEVLVWRFDSKLAFHHMARELHPEYYKDC

Nef-O

MGGKWSKRSMGGWSAIR**QRLLRARG**EPRAEPAADGVGAVSRDLEKHGAITSSNTAATNAD
CAWLEAQEEEEVGFPVRPQVPLRPMTYK**L**ALD**M**SHF**I**KEKGGLEG**IYY**S**A**RR**HR**ILD**IYL**
EKEEG**II**PDWQNYTPGPGIRYPLTFGWCFKLVPVEPEKVEEANEGENNSLLHPMSLHGME
DAEKEVLVWRFDSKLAFHHMARELHPEYYKDC

Nef-P

MGGKWSKRSMGGWSAIRERMRRAE**TYGRLLGEVE**DGVGAVSRDLEKHGAITSSNTAATNA
DCAWLEAQEEEEVGFPVRPQVPLRPMTYK**L**ALD**M**SHF**I**KEKGGLEG**IYY**S**A**RR**HR**ILD**IY**
LEKEEG**II**PDWQNYTPGPGIRYPLTFGWCFKLVPVEPEKVEEANEGENNSLLHPMSLHGM
EDAEKEVLVWRFDSKLAFHHMARELHPEYYKDC

Nef-Q

MGGAISMRRSRPSGDLRQRLLRARGEPRAEPAADGVGAVSRDLEKHGAITSSNTAATNAD
CAWLEAQEEEEVGFPVRPQVPLRPMTYK**L**ALD**M**SHF**I**KEKGGLEG**IYY**S**A**RR**HR**ILD**IYL**
EKEEG**II**PDWQNYTPGPGIRYPLTFGWCFKLVPVEPEKVEEANEGENNSLLHPMSLHGME
DAEKEVLVWRFDSKLAFHHMARELHPEYYKDC

Nef-R

MGGKWSKRSMGGWSAIR**QRLLRARGETYGRLLGEVE**DGVGAVSRDLEKHGAITSSNTAAT
NADCAWLEAQEEEEVGFPVRPQVPLRPMTYK**L**ALD**M**SHF**I**KEKGGLEG**IYY**S**A**RR**HR**ILD
IYLEKEEG**II**PDWQNYTPGPGIRYPLTFGWCFKLVPVEPEKVEEANEGENNSLLHPMSLH
GMEDAEKEVLVWRFDSKLAFHHMARELHPEYYKDC

Nef-S

MGGKWSKRSMGGWSAIRERMRRAE**TAGRAAGEVE**DGVGAVSRDLEKHGAITSSNTAATNA
DCAWLEAQEEEEVGFPVRPQVPLRPMTYK**L**ALD**M**SHF**I**KEKGGLEG**IYY**S**A**RR**HR**ILD**IY**
LEKEEG**II**PDWQNYTPGPGIRYPLTFGWCFKLVPVEPEKVEEANEGENNSLLHPMSLHGM
EDAEKEVLVWRFDSKLAFHHMARELHPEYYKDC

Nef-T

MGGKWSKRSMGGWSAIRERMRRAE**TYGRLLGEVE**DGVGAVSRDLEKHGAITSSNTAATNA
DCAWLEAQEEEEVGFPVRPQVPLRPMTYK**L**ALD**M**SHF**I**KEKGGLEG**IYY**S**A**RR**HR**ILD**IY**
LEKEEG**II**PDWQNYTPGPGIRYPLTFGWCFKLVPVEPEKVEEANEG**A**NNS**AA**HPMSLHGM
EDAEKEVLVWRFDSKLAFHHMARELHPEYYKDC
